# Kainate-induced ATP release contributes to spinal excitotoxicity that is attenuated by purinergic blockade

**DOI:** 10.3389/fneur.2026.1911699

**Published:** 2026-09-22

**Authors:** Benjamín Zylberberg, M. Florencia Coronel, Graciela L. Mazzone

**Affiliations:** 1 Instituto de Investigaciones en Medicina Traslacional (IIMT), CONICET-Universidad Austral, Pilar, Buenos Aires, Argentina; 2 Facultad de Ciencias Biomédicas, Universidad Austral, Pilar, Buenos Aires, Argentina

**Keywords:** ATP release, glutamate release, P2X receptors, purinergic antagonist, spinal cord injury

## Abstract

**Introduction:**

Severe spinal cord injury (SCI) is characterized by irreversible neuronal loss and permanent motor/sensory dysfunction with very limited treatment. This study aimed to assess the role of purinergic signalling at a very early stage of SCI following focal chemical injury induced by kainate (KA).

**Methods:**

Male and female C57BL/6J and BALB/c mice were used in a SCI model. Real-time biosensor recordings were employed to monitor glutamate and ATP release from spinal tissue under in vitro conditions simulating SCI. Changes in purinergic receptor expression, neuronal survival, glial activation, and locomotor function were evaluated, including the effects of the purinergic antagonist Brilliant Blue G (BBG).

**Results:**

KA treatment induced a marked increase in extracellular glutamate and ATP release and significantly elevated mRNA levels of P2X4 and P2X7 receptors. BBG treatment significantly increased neuronal survival compared with KA-treated tissues. No effects on astrocyte or microglial activation were detected at this early stage. In vivo, KA markedly impaired locomotor function, whereas BBG co-treatment produced partial functional recovery.

**Discussion:**

These findings support a role for early ATP release in modulating neuronal survival following excitotoxic injury, potentially through CREB-dependent signalling pathways. ATP-dependent mechanisms may therefore represent promising targets for future neuroprotective strategies in SCI.

## Graphical abstract

**Figure:**
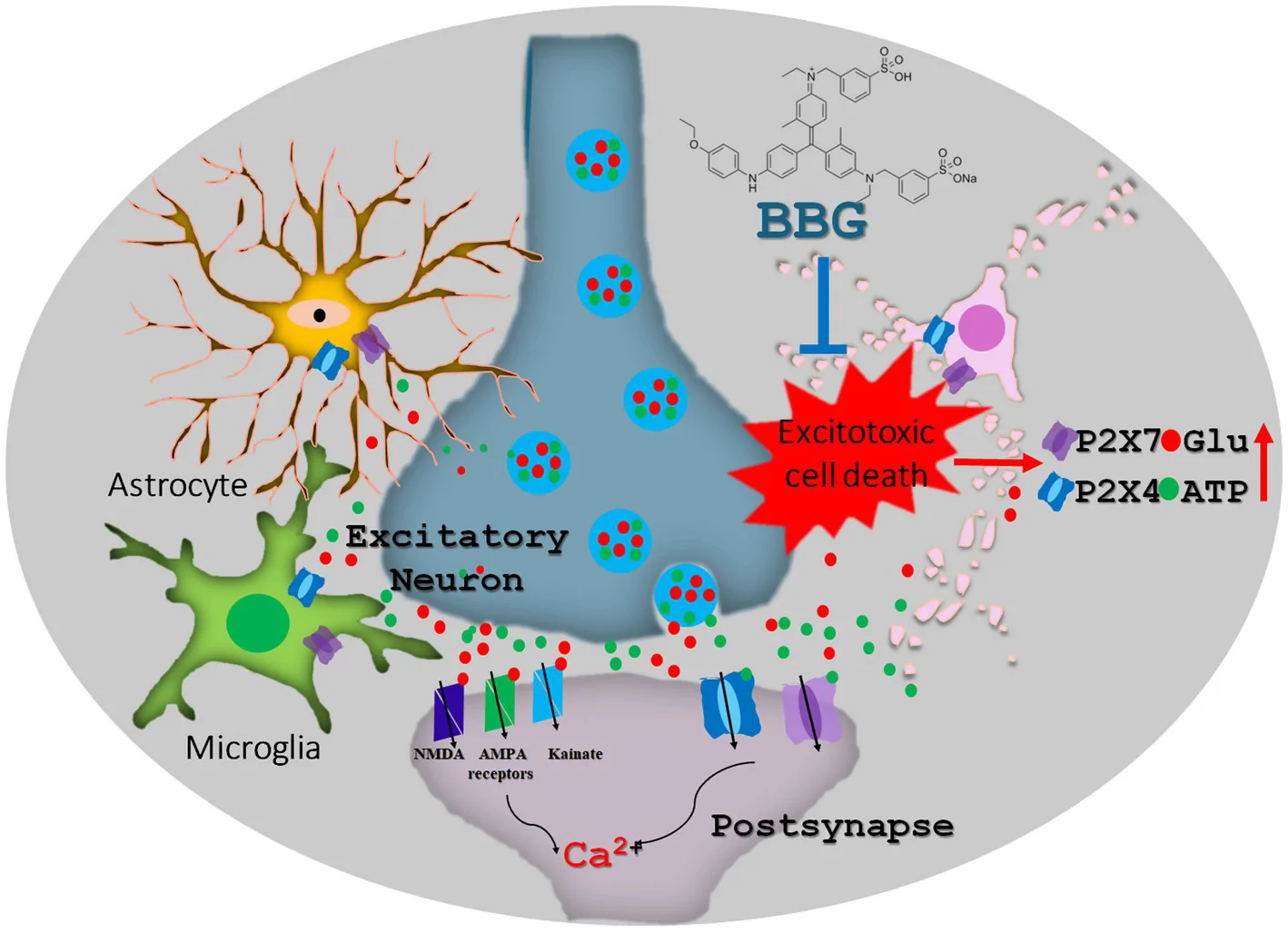

## 1 Introduction

Spinal cord injury (SCI) is a highly debilitating disorder affecting mainly young individuals, causing profound and long-term impairments through primary trauma and subsequent secondary mechanisms that impact functions below the level of injury (1, 2). The initial injury consists of direct mechanical trauma to the spinal cord, resulting in segmental damage marked by axonal disruption, contusion, hemorrhage, ischemia, and tissue swelling (3, 4). Temporally, after the primary injury, subsequent processes generate secondary damage that markedly increases the loss of surrounding tissue, mainly through excitotoxicity driven by excessive glutamate release (5, 6) and by neuroinflammation, largely triggered by the release of adenosine triphosphate (ATP) (7). Disruption of ATP homeostasis consequently impairs mitochondrial function, leading to further dysregulation of ATP production (8, 9) and accelerating the secondary injury cascade, which amplifies neuronal death and tissue degeneration (10). Therefore, therapeutic strategies that preserve or restore ATP production during the acute phase of SCI could stabilize mitochondrial function, limit oxidative damage, and prevent neuronal apoptosis (11), thereby improving long-term neurological and functional recovery.

At later stages of SCI, in the inflammatory microenvironment, ATP acts as a key extracellular signalling molecule that regulates diverse cellular processes by activation of P2 receptor families (12). ATP released from injured or stressed cells activates purinergic P2X and P2Y receptors in the spinal cord, contributing to excitatory postsynaptic responses (13). Indeed, activation of these receptors by extracellular ATP contributes to persistent hypersensitivity and amplifies inflammatory cascades (14), thereby linking energy failure to maladaptive neuronal signalling after SCI (7). Furthermore, purine nucleosides and nucleotides released following cell injury act as paracrine mediators through specific membrane receptors, thereby modulating both inflammatory and neuroimmune responses (15). Therapeutic strategies that restore ATP homeostasis or modulate P2X/P2Y receptor activity could therefore simultaneously preserve mitochondrial function, limit secondary injury–related functional impairments, such as deficits in respiratory and motor plasticity, following SCI (16), highlighting ATP’s dual role as both an energy substrate and a key neuromodulator in injury and recovery (17).

The main purpose of this study was to elucidate the secondary events associated with SCI mediated by purinergic receptors by using BBG, a non-nucleotide purinergic antagonist, after an acute excitotoxic injury. We hypothesize that the release of ATP and glutamate acts as a time-sensitive determinant of neuronal survival, and that timely antagonism of ATP-driven signalling cascades can stabilize the injury environment, providing potentially useful insights for the rational use of selective purinergic modulators. Understanding the interplay between different effectors and the critical therapeutic window is essential to design strategies that mitigate secondary damage induced by excitotoxicity and limit locomotor impairment at the earliest possible stage. Therefore, we investigated the role of purinergic signalling, specifically ATP-related release, following chemically induced SCI and its contribution to motor dysfunction.

## 2 Materials and methods

### 2.1 Animals

C57BL/6J mouse embryos were used for organotypic spinal cord slices preparation, following guidelines from the Ethical Committee of the Scuola Internazionale Superiore di Studi Avanzati (Trieste), NIH, and Italian law D.Lgs. 27/1/92 n.116 (European directives 86/609 and 93/88). Adult and early postnatal BALB/c mice were sourced from the Animal Resource Facility (School of Biomedical Sciences, Austral University). All procedures were conducted in accordance with the local Animal Care Committee (protocols N° 2018-02, 2019-05, 2021-08, and 2024-2) following animals ethical guidelines provided by the School of Biomedical Sciences of Austral University. All procedures were conducted to minimize animal use and distress. Adult BALB/c mice were bred in our animal facility and maintained under a 12-h light/dark cycle, housed in groups of 2–3 per cage on corncob bedding, with food and water provided ad libitum.

### 2.2 Mouse organotypic cultures and isolated spinal cord preparations

Organotypic spinal cord slice cultures were prepared using C57BL/6J mouse embryos at 13 days of gestation, adhering to our established protocol as outlined in previous reports (5). Briefly, fetuses were obtained by caesarean section from timed-pregnant mice euthanized by gradual CO₂ exposure. Tissue slices were cultured for 22 days *in vitro* in a medium containing 82% Dulbecco’s Modified Eagle Medium (osmolarity 300 mOsm, pH 7.35), 8% sterile water, 10% fetal bovine serum (FBS Invitrogen, Carlsbad, CA, USA), and 5 ng/mL nerve growth factor (NGF; Alomone Laboratories; Jerusalem, Israel). Spinal cord preparations were obtained *in vitro* from neonatal (postnatal days 0–3) male and female BALB/c mice, following previously established procedures (18). Briefly, after decapitation, the entire spinal cord was carefully dissected and transferred to oxygenated Krebs solution (in mM: 113 NaCl, 4.5 KCl, 1 MgCl₂·7H₂O, 2 CaCl₂, 1 NaH₂PO₄, 1 HEPES, 11 glucose), continuously gassed with 100% O₂.

### 2.3 Experimental protocol for *in vitro* excitotoxic SCI

To model excitotoxic insult, isolated spinal cord preparations were exposed to kainic acid (KA, 100 μM, N = 9, Figure 1A) for 1 h, followed by 3 h recovery period in Krebs solution (control group (*N* = 12). To investigate the effects of BBG (10 μM, AppliChem, CI 42655, Barcelona, Spain)), 10 μM BBG was applied alone or together with KA (for 1 h), followed by BBG alone for 3 h (represented as BBG (*N* = 3, given the limited sample size, results from this group should be interpreted with caution) or KA + BBG 4 h (*N* = 5), respectively) and maintained for the following 3 h with BBG in Krebs solution, respectively. Spinal cords were maintained in Krebs solution for 4 h before gene expression and histological analyses (*N* = 29, where N is the total number of mice used in the study).

**Figure 1:**
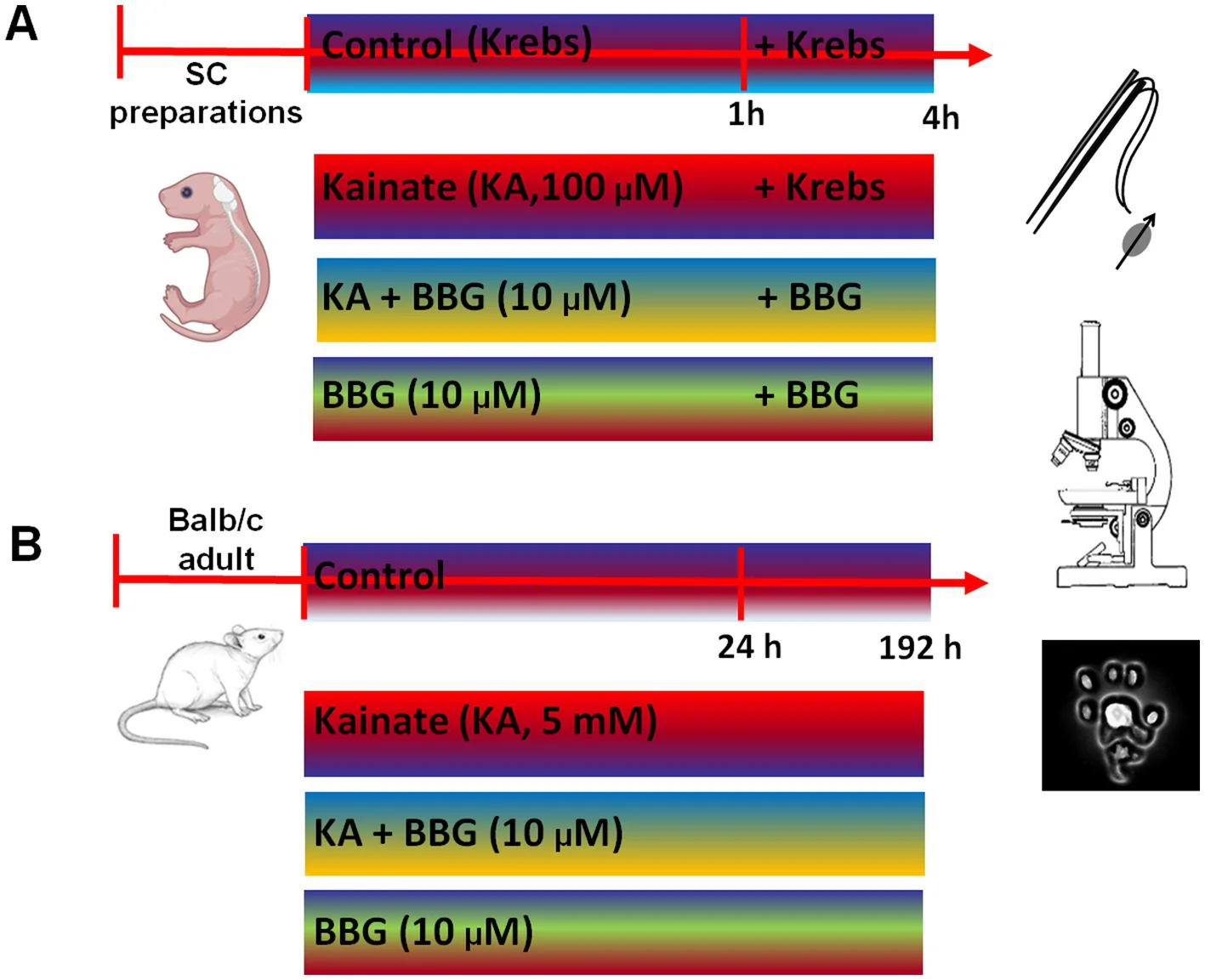
Protocols of drug application followed by electrochemical, molecular, immunohistochemical and behavioral studies performed either *in vitro* using slice cultures **(A)** or spinal cord preparations or *in vivo* using adult BALB/c mice **(B)**.

### 2.4 *In vivo* chemical lesion model of the spinal cord

Spinal cord lesions were induced in adult male BALB/c mice (8–10 weeks old, 20–30 g) via intraspinal injection of 1 μL of 5 mM KA (Figure 1B). Mice were anesthetized with 2% isoflurane (Piramal Healthcare Ltd., Buenos Aires, Argentina) in oxygen using a vaporizer (Rockmart GA, HERLAM Laboratory, Buenos Aires, Argentina) to replicate excitotoxic damage. Anesthesia depth was assessed using paw and tail pinch reflexes. A dorsal laminectomy was performed at thoracic levels T10–T12, followed by intraspinal injection of KA (19). Tramadol hydrochloride (100 mg; 5 mg/kg, BAGO, Buenos Aires, Argentina) was administered subcutaneously every 12 h for 3 days to alleviate pain following the spinal injury. Recovery cages were prepared with soft food available *ad libitum* and water bottles with long sipping tubes. The animals maintained voluntary urination. To investigate BBG effects on spinal locomotor networks 27 mice were used in the study (Figure 1B; control group: *N* = 11; KA group: *N* = 7). BBG (45 mg/Kg, IP) was applied alone (*N* = 5) or immediately following KA (KA + BBG, *N* = 5). At the conclusion of all experimental procedures, mice were euthanized in a chamber using 100% carbon dioxide (CO₂) at a fill rate of 30–70% of the chamber volume per minute, in accordance with established guidelines to ensure irreversible euthanasia (20).

### 2.5 Electrochemical release

Electrochemical recordings were performed during KA application for 1 h (100 μM, *N* = 5, Figure 1A) in organotypic spinal cord slice cultures. Electrochemical biosensors (Sarissa Biomedical Ltd., Coventry, UK) were utilized in accordance with our prior report (6). Real-time recordings were acquired *via* a potentiostat (Pinnacle Technology Inc., Lawrence, KS, USA) and analysed offline using PAL software (v1.6.7; Pinnacle Technology Inc.). Glutamate- and ATP-sensitive biosensors, together with control (null) sensors, were located bilaterally adjacent to the ventral fissure in organotypic spinal cord slice cultures. Glutamate levels were measured using a glutamate-selective electrode, while ATP release was simultaneously recorded with an ATP-selective electrode within the same slice. Sensor specificity was confirmed by calibration curves for glutamate (0.5–50 μM) and ATP (0.5–100 μM). Background currents were determined using the null electrode. Recordings from all electrodes were performed in a basal solution containing (in mM): NaCl 152, KCl 5, CaCl₂ 2, MgCl₂ 1, HEPES 10, and glucose 10, adjusted to pH 7.4 and an osmolarity of 300–320 mOsm (Sigma–Aldrich, Milan, Italy).

### 2.6 Quantitative real-time PCR analysis

The expression levels of P2X4 and P2X7 were assessed *in vitro* spinal cord preparations from neonatal BALB/c mice using real-time reverse transcription PCR (RT-PCR). Gene-specific primers for the detection of P2X4 and P2X7 (Table 1) were designed using Primer-BLAST.^1^ Total RNA was isolated using Tri Reagent solution, following the manufacturer’s protocol (Invitrogen). RNA concentrations were measured at 260 nm using a spectrophotometer. For reverse transcription, 1 μg of total RNA was processed with the iScript cDNA Synthesis Kit (Bio-Rad, Cat. No. 170-8,891) according to the manufacturer’s instructions. The thermocycler settings were 25 °C for 5 min, 42 °C for 45 min, and 85 °C for 5 min. Quantitative real-time PCR was performed using the AriaMx Real-Time PCR System (Agilent Technologies) with SYBR Green dye (SYBR Green Supermix kit IQ™; Bio-Rad). A melting curve analysis confirmed the specificity of the PCR products. Relative mRNA transcript levels were calculated using the comparative CT method [ΔΔCT (21)] and normalized to the β-actin, housekeeping gene, as previously described (22).

**Table 1:** Primer sequences used for real-time RT-PCR analysis.

Gene name	Primer forward	Primer reverse
P2X4	CTCATCCGCAGCCGTAAAGT	ACGAACACCCACCCAATGAC
P2X7	GCACGAATTATGGCACCGTC	CCCCACCCTCTGTGACATTCT
β-actin	CCTTCTTGGGTATGGAATCCTGTG	CAGCACTGTGTTGGCATAGAGG

### 2.7 Immunostaining and cell counting in slice preparations

Spinal cords from both *in vitro* and *in vivo* experiments were fixed overnight in 4% paraformaldehyde (PFA) and subsequently cryoprotected in 30% (w/v) sucrose, as previously described (18). Briefly, 20 μm transverse spinal cord sections (T13–L5) were obtained using a microtome. For immunofluorescence, sections were incubated in blocking solution (5% fetal bovine serum, FBS or normal goat serum, NGS; 5% bovine serum albumin, BSA; 0.3% Triton X-100; 1xPBS) for 1 h at room temperature and then incubated with primary antibodies: NeuN (1:200; Merck Millipore, Milan, Italy; MAB377) for neurons, S100β (1:750; Dako, Glostrup, Denmark; Z0311) for astrocytes, Iba1 (1:500 or 1:800; Wako, Osaka, Japan, 019-19,741 or Abcam, Miami, USA, ab178847) for microglia and CREB (rabbit monoclonal; 1:500; Cell Signaling Technology, Inc., Leiden, The Netherlands; D76D11) for endogenous levels of total CREB protein. Primary antibodies were visualized using the corresponding secondary fluorescent antibody (1:200; Jackson ImmunoResearch Laboratories, Inc., Pennsylvania, USA, such as goat anti-mouse (115-095-003); donkey anti-rabbit (711-025-152). Cell nuclei were counterstained with 4′, 6-diamidino-2-phenylindole (DAPI, 1 μg/mL). NeuN-positive cells were quantified from 20x images acquired with a Nis-Eclipse microscope (NIKON, Amsterdam, Netherlands). Cell counts were performed in two regions of interest (dorsal and ventral) using ImageJ software (NIH, https://imagej.nih.gov/ij/index.html). S100β and Iba1 immunofluorescent signal was quantified by densitometric analysis in 387×387 μm^2^ fields using ImageJ software, and results were expressed in arbitrary units (AU). CREB-positive cells were quantified by manual scoring of 20 × magnification images within fields measuring 390 × 390 μm^2^) using ImageJ software. In addition, nuclear localisation and distribution of the CREB immunofluorescence signal (AU), together with DAPI staining, were analysed using ImageJ software.

### 2.8 Behavioral assessment: open field locomotion

Hindlimb locomotor function was assessed using the Basso Mouse Scale (BMS) in a square open field (40 × 40 cm), evaluating both right and left hindlimb movements over a 5 min period, as previously described (19). Evaluations were conducted pre-injury to ensure normal functional status, at 24 h post-surgery, and at 1 week after injury by at least two independent experimenters. Scoring was performed using the 9-point BMS scale (23), supported by an open-source software tool (24) to standardise and asses the effects of the different experimental conditions.

### 2.9 Drugs

Experimental drugs were acquired from Sigma-Aldrich (St. Louis, MO, USA), whereas kainic acid (KA) was purchased from Tocris Bioscience (Buenos Aires, Argentina; Cat. No. 0222). DMEM/High Glucose, penicillin, and streptomycin were obtained from Euroclone (Paignton, UK), chicken plasma from Rockland Immunochemicals (Gilbertsville, PA, USA), and thrombin from Merck KGaA (Darmstadt, Germany).

### 2.10 Statistics

Statistical analyses were carried out using GraphPad Prism (version 8; GraphPad Software, San Diego, CA, USA), and data are reported as mean ± SEM. “*N*” indicates the number of animals or neonatal spinal cords, while “*n*” refers to the number of spinal cord slices. Normality was assessed with the Shapiro–Wilk test, and no outlier tests were conducted. One-way or two-way analysis of variance (ANOVA) was used for multiple group comparisons of parametric data, followed by Tukey’s or Dunnett’s *post hoc* tests. The Kruskal–Wallis test was applied for non-parametric data with appropriate multiple comparisons. For two independent groups, parametric data were evaluated using Student’s *t*-test or the Mann–Whitney test, with statistical significance set at *p* < 0.05.

## 3 Results

### 3.1 KA induced the release of glutamate and ATP and the transcriptional upregulation of P2X4 and P2X7 receptors

Our prior findings indicate that kainate exposure causes pronounced neuronal depolarization, followed by neuronal loss in spinal cord and organotypic cultures (5, 25), associated with a strong release of endogenous glutamate (6). In the present study we investigated whether the glutamate peak induced by 100 μM KA was coincident with ATP release. Figure 2A shows that KA evoked the release of endogenous glutamate and also ATP. On the basis of the calibration tests, Figure 2B shows that the peak current amplitude corresponded to 1.66 ± 0.16 μM extracellular glutamate and 36.65 ± 11.40 μM ATP. Since these observations demonstrated that ATP was correleased with glutamate, we speculated that purinergic signals might have a role in the initial excitotoxic stimulus, probably affecting neuronal survival. To confirm the involvement of ATP signalling, we evaluated the expression of P2X4 and P2X7 following KA treatment at *in vitro* neonatal spinal cord preparations. Figures 2C,D show that after kainate application, P2X4 and P2X7 mRNA levels significantly increased after 4 h, suggesting purinergic signalling involvement in KA excitotoxicicity.

**Figure 2:**
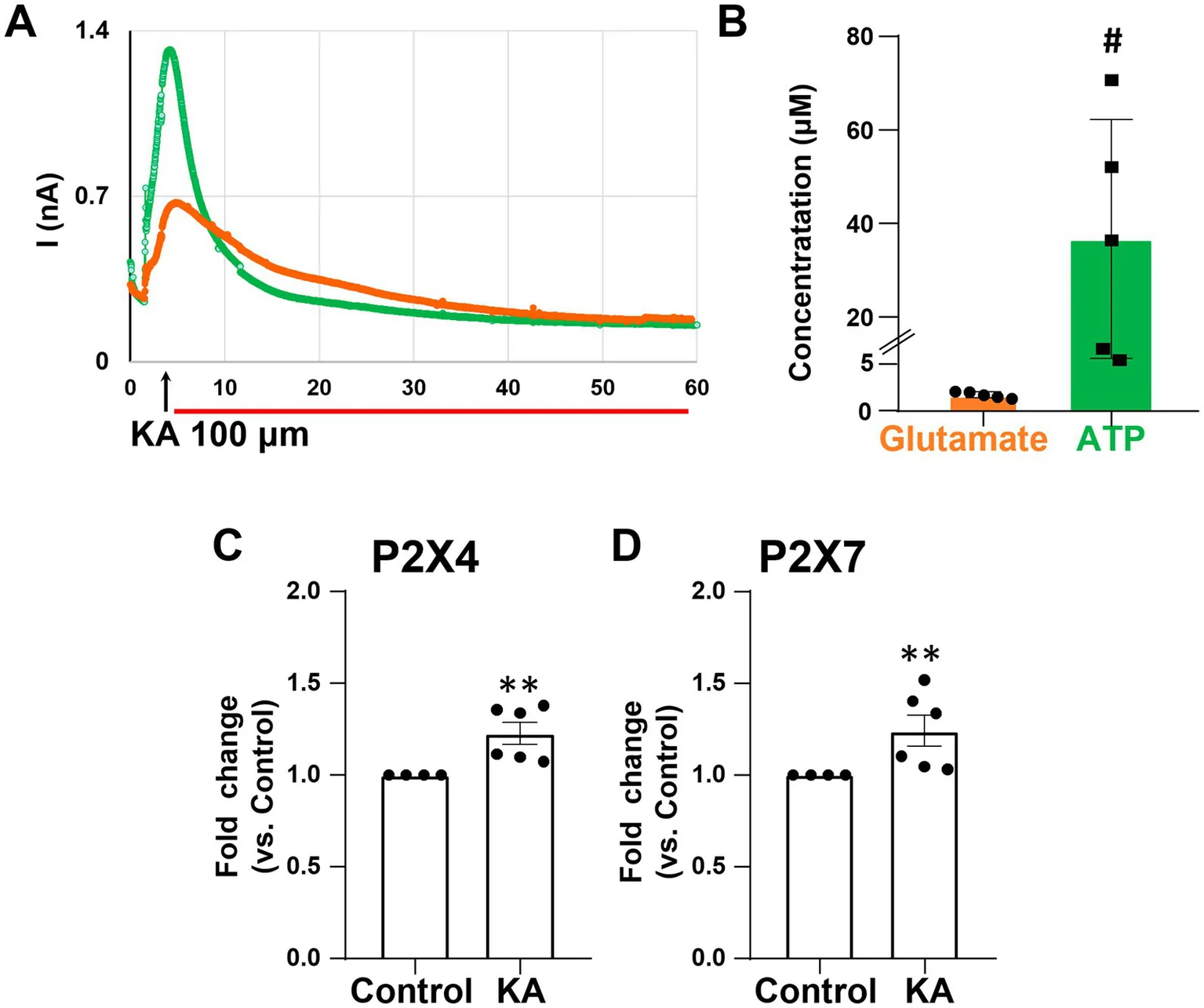
KA-induced ATP release and transcriptional modulation of purinergic receptors in spinal cord *in vitro* models: **(A)** representative slice that was exposed to KA for 1 h, while the release of ATP (orange) and glutamate (green) was continuously monitored. The ordinate depicts the average redox current over time throughout the experiment (*n* = 4–5 slices exposed to KA). **(B)** Histograms show the average mean concentration of ATP and glutamate released during KA application for 1 h, *n* = 5 slices, *p* = 0.0304, *t*-test. **(C)** P2X4 and **(D)** P2X7 mRNA levels were quantified by RT-PCR and reported as relative changes compared to controls. Amplification data were normalized to β-actin mRNA levels for each gene analysed, *N* = 4–6, **p ≤ 0.01, vs. control; Mann–Whitney test.

### 3.2 BBG protected cultures against neuronal cell death

To further investigate whether ATP release and purinergic signalling can modulate cell death during KA-induced excitotoxic injury, we examined the effect of BBG treatment on the survival of spinal cord preparations. Thus, we assessed BBG impact on pyknotic cell death, a well-established marker used in our previous studies. As expected, Figure 3A shows the typically increased nuclear condensation in dorsal and ventral spinal cord regions after KA treatment, consistent with previous reports in rat spinal cord preparations (5). Indeed, KA treatment resulted in an increased percentage of pyknotic nuclei in dorsal and ventral spinal cord regions (Figure 3C), confirming earlier observations (5). On the contrary, while BBG alone did not induce any changes in this marker, application of KA + BBG prevented cell death, both in dorsal and ventral regions (Figures 3A,D). Analysis of the cell types affected by KA treatment revealed that the number of NeuN-positive neuronal cells was reduced after KA administration (Figure 3B), in agreement with the neuronal loss previously reported following KA exposure (5). Histograms show that cell death correlated with neuronal loss in dorsal and ventral regions, which was preserved by BBG application (Figure 3D). Thus, we could speculate that after the excitotoxic stimulus, ATP exacerbates neuronal cell death, at least in part *via* ATP receptors, which can be antagonized by BBG effects.

**Figure 3:**
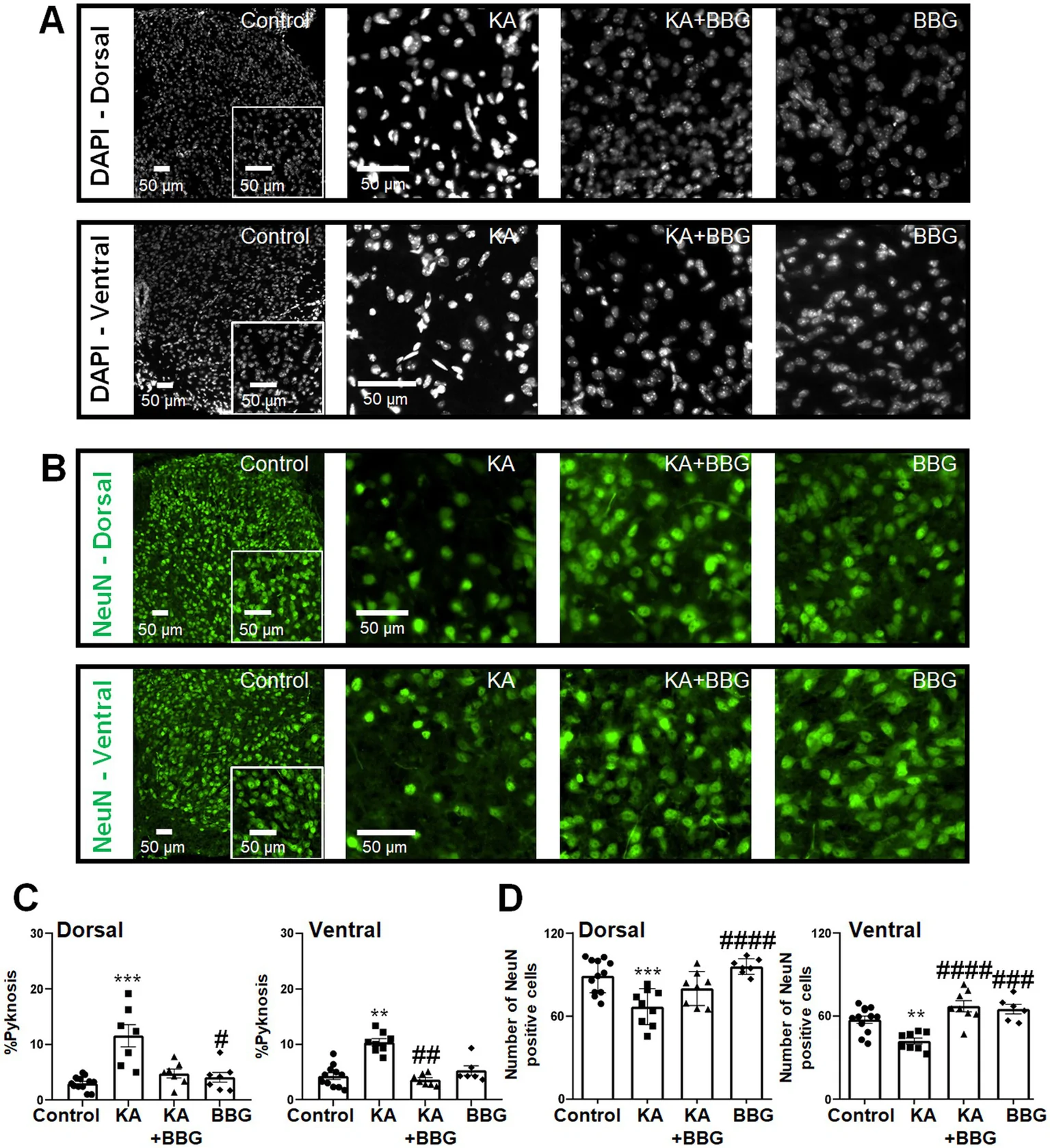
KA-induced pyknosis and neuronal loss are mitigated by BBG: **(A)** representative DAPI-labelled cells in dorsal and ventral areas of the spinal cord in control conditions, after KA, KA + BBG or BBG application; **(B)** examples of the dorsal and ventral region with NeuN positive neurons (green); **(C)** Histograms showing average percentage of pyknotic cells in the dorsal and ventral spinal cord region, *n* = 6–12, Kruskal–Wallis test D: *p* = 0.0008—V: *p* = 0.0005, **p < 0.01, ***p < 0.001 vs. control, #*p* < 0.05, ##*p* < 0.01 vs. KA; multiple comparisons by the Dunnett test; **(D)** Histograms representing the mean number of NeuN-positive cells in both regions examined, *n* = 6–12, one-way ANOVA test, *n* = 6–12, D: *F* = 10.28—V: *F* = 12.15, ***p < 0.001 vs. control, ###*p* < 0.001, ####*p* < 0.0001 vs. KA, multiple comparisons by the Tukey’s multiple comparisons test.

### 3.3 BBG: unchanged glial phenotypes but potential CREB-driven neuronal effects

Even though our earlier work was conducted in rat organotypic spinal cord slice cultures, we did not observe any apparent effect of KA on astrocytes and microglia at early time points (26), we decided to examine whether KA affected these cell types in a mouse model and, if so, whether BBG could modulate their response. We therefore performed histological analyses of astrocytes (Figure 4A) and microglia (Figure 4B). These experiments indicate that the expression of the glial markers S100β (Figure 4C) and Iba1 (Figure 4D) remained unchanged after KA treatment. While this suggests that KA does not strongly affect these markers at early time points, other aspects of glial activation cannot be ruled out.

**Figure 4:**
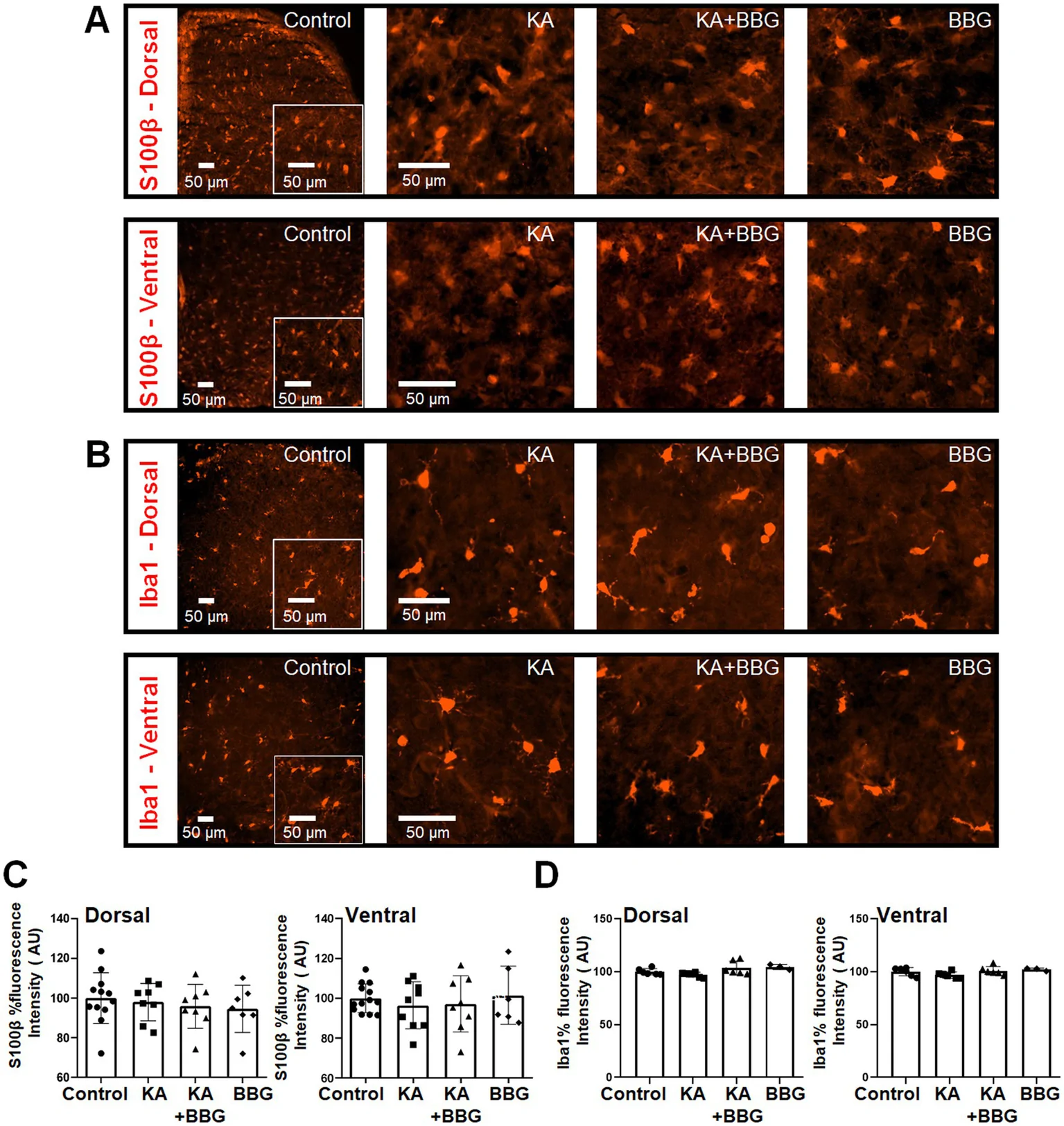
Expression of glial and microglial markers remains unchanged following treatments: **(A)** examples of representative S100β staining (red) in control condition, after KA, KA + BBG or BBG application; **(B)** representative staining of Iba1 positive cells (red) at dorsal and ventral spinal cord regions; **(C)** histograms representing S100β mean percentage of fluorescence intensity (AU) in the dorsal and ventral spinal cord regions (*n* = 6–13, Kruskal–Wallis test, *ns*); **(D)** Quantitative analysis presented as histograms of Iba1 mean percentage of fluorescence intensity (AU) after KA, KA + BBG or BBG application (*n* = 6–13, Kruskal–Wallis test, *ns*). Note that no significant differences were found for S100β and/or Iba1 following KA and/or BBG treatments.

Our previous study identified potential early targets downstream of glutamate release, such as CREB (27), which may have significant implications for the development of effective treatments. In this context, we propose that BBG treatment following KA application may modulate excitotoxicity, particularly in the ventral area (Figure 5A), indicating a probable link between this transcription factor and neuronal cell loss. In our experiments, KA exposure significantly reduced the number of nuclei positive for CREB, an effect that was markedly reversed by BBG co-treatment (Figure 5B). Representative CREB immunofluorescence patterns in ventral spinal cord cells under the different experimental conditions are shown in Figures 5A,C. Line-scan fluorescence intensity analysis corresponding to the cell indicated by the dashed bar (Figures 5A,C) is shown on the right, demonstrating a reduction in nuclear CREB immunofluorescence intensity (expressed in arbitrary units, AU) under KA treatment, whereas BBG co-treatment restored CREB nuclear signal levels comparable to control conditions (Figure 5D). No differences in DAPI staining were observed among experimental and control groups (Figure 5E).

**Figure 5:**
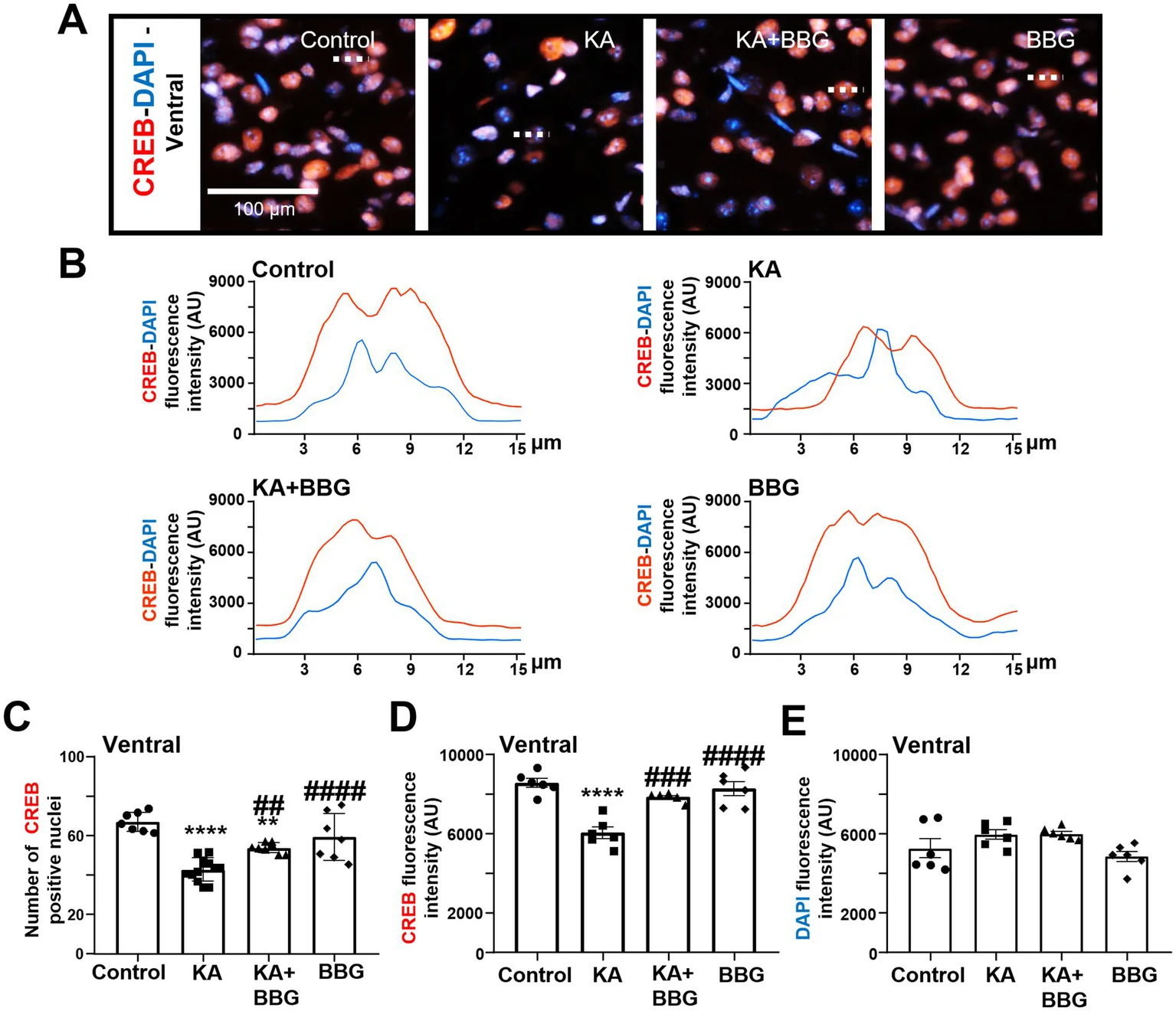
BBG appears to reverse the nuclear translocation of CREB that is induced by KA. **(A)** Examples of representative CREB staining (red) in DAPI-stained cells (blue) in control condition, after KA, KA + BBG or BBG application at ventral spinal cord region **(B)** Mean values of CREB-positive nuclei in ventral regions presented as histograms (*n* = 7–13, one-way ANOVA test, *F* = 21.32, ^****^p < 0.0001, ^**^p < 0.01 vs. control, ^##^*p* < 0.01, ^####^*p* < 0.0001 vs. KA, multiple comparisons by the Tukey’s multiple comparisons test.) **(C)** Representative line-scan fluorescence intensity profiles for CREB and DAPI signals, expressed in arbitrary units (AU) per μm, across the different experimental groups **(D,E)** Histograms showing the maxima fluorescence intensity for CREB (*n* = 6, one-way ANOVA test, *F* = 19.45, ^****^p < 0.0001, ^###^*p* < 0.001, ^####^*p* < 0.0001 vs. KA, multiple comparisons by the Tukey’s multiple comparisons test.) and DAPI signals (*n* = 6, one-way ANOVA test, *F* = 3.318, ns in ventral spinal cord regions).

### 3.4 Locomotor outcomes after *in vivo* KA excitotoxicity with BBG treatment

Studying the timing of delayed effects following SCI is a complex task. *In vitro* studies can only be conducted over a short period of hours or days (18), limiting their usefulness. Therefore, animal studies are typically the most suitable approach for conducting repeated neuropathological sampling and also examining locomotor behavior. In these experiments we observed that SCI animals exhibited a significant reduction in the BMS score at both 1 and 8 days post-injury (dpi, Figure 6), with a marked functional loss in both the left (Figure 6A) and right (Figure 6B) hind limbs. On the contrary, KA-injected animals that received BBG at 8 dpi showed similar values to those detected in the control group. However, statistical evaluation did not identify significant differences between KA and KA + BBG groups for either the left or right hind limbs (Figure 6).

**Figure 6:**
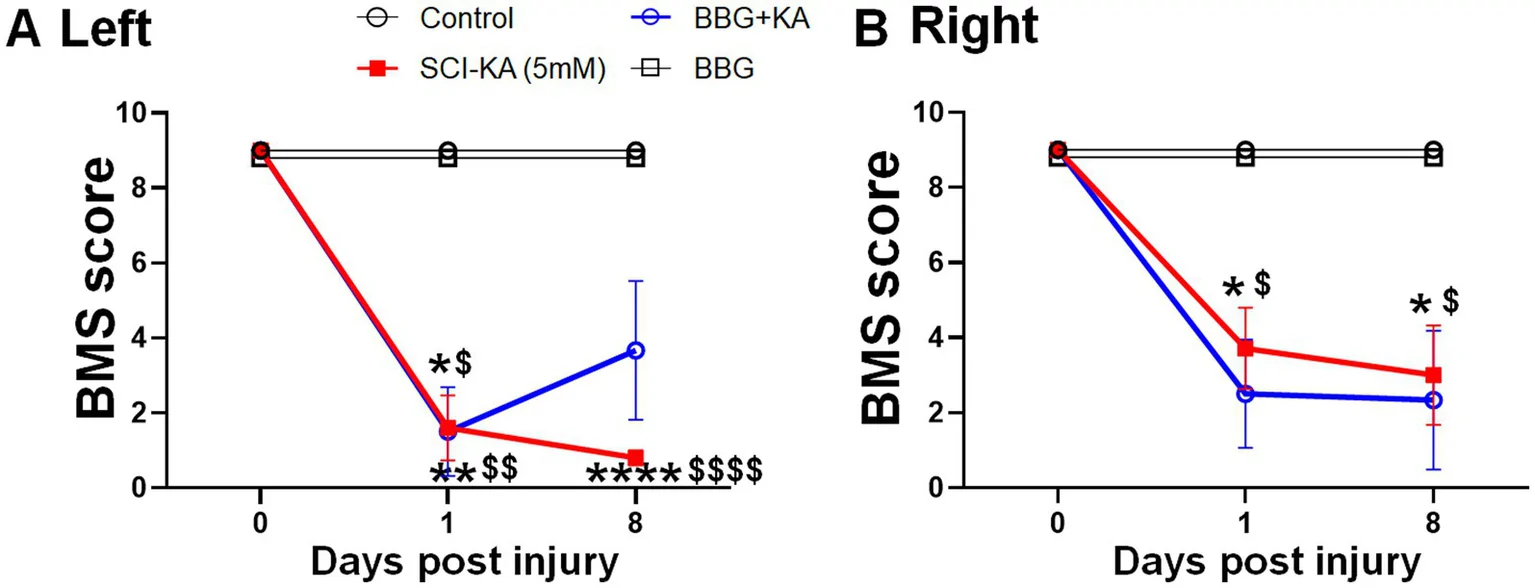
Effects of BBG on motor activity following chemical spinal cord lesion. BMS scores were determined before and after KA administration (1 or 8 dpi), with or without BBG, for both left **(A)** and right **(B)** hindlimbs during locomotion. *N* = 5–11 Two-way ANOVA, L: *F* (1.847, 37.86) = 129.5—R: *F* (1.823, 41.02) = 34.49: ^*^p < 0.05, ^**^p < 0.01, ^****^p < 0.0001 vs. control, ^$^*p* < 0.05, ^$$^*p* < 0.01, ^$$$$^*p* < 0.0001 vs. BBG; multiple comparisons by Tukey’s multiple comparisons test.

## 4 Discussion

The present study reveals that early release of ATP, alongside glutamate release and following excitotoxic damage, may influence neuronal survival. Additionally, we demonstrated that antagonizing ATP signalling pathway using BBG attenuates cell death and might potentially improve locomotion, probably by increasing network plasticity. By combining *in vitro* and *in vivo* spinal cord injury models, we addressed both acute injury mechanisms and chronic functional outcomes. Together, these findings uncover a previously underappreciated contribution of purinergic signalling to early injury progression.

### 4.1 ATP was early released along with glutamate after KA-induced excitotoxicity

Using an *in vitro* SCI model of organotypic spinal cord cultures, our previous work demonstrated that KA (0.1–0.5 μM) can induce a rapid and robust increase in extracellular glutamate levels (6) and lactate dehydrogenase release (5), both occurring within a limited timeframe (1 h of KA exposure followed by 3 h of washout). These early events are essential for understanding the initial cascade triggered by KA. This experimental design allowed us to focus on mechanisms preceding extensive cell death and ATP dynamics related to secondary injury, as documented in our earlier studies, which also examined their impact on neuronal survival and potential modulation of network plasticity. Recognizing that some events can occur at later time points, such as glial phenotypic modulation (26), our recent investigations have decisively incorporated *in vivo* experiments, complemented by behavioral assessments and extended observation periods. This approach allowed us to uncover previously uncharacterized mechanisms underlying purinergic-dependent secondary damage, providing new evidence on how blocking specific purinergic pathways can modulate early post-injury dynamics. Building on these findings, the present study sought to identify the cellular targets affected after spinal injury and to elucidate the cellular targets through which ATP, in concert with glutamate, contributes to the pathophysiology of chemical insults.

Recent evidence from experimental models of SCI indicates that extracellular ATP increases after injury and contributes to secondary neuronal damage (28). Consistent with these observations, our results demonstrate that KA treatment markedly increases early ATP release concomitantly with glutamate and is associated with transcriptional upregulation of P2X4 and P2X7 receptors. Given the central role of extracellular ATP in neuronal communication under both physiological and pathological conditions (29), these findings support the notion that purinergic signalling contributes to the early excitotoxic cascade triggered by KA. Here, we used enzyme-based biosensors to measure the real-time and spatiotemporal dynamics of ATP release following chemical spinal injury. The sensors were positioned near, but not within, the ventral spinal fissure tissue, and therefore are likely to detect the accumulated ATP originating from multiple sources, which is probably released *via* different channels, such as pannexins, connexins, and calcium homeostasis modulators, through multiple gating mechanisms (30). Beyond its role as a damage-associated signal released by injured cells, recent evidence suggests that ATP may contribute to the encoding and propagation of injury-related information through glial communication networks. Nevertheless, our experimental approach does not allow identification of the cellular sources of ATP release, nor does it exclude the contribution of additional signalling pathways that may cooperate with ATP in coordinating responses to tissue injury (31).

### 4.2 Purinergic antagonism prevented spinal neuronal loss and partially improved motor recovery

Under physiological conditions, purinergic signalling plays a crucial role in fast excitatory transmission by generating excitatory postsynaptic responses (12) and regulating ionic homeostasis, particularly sodium (Na^+^), potassium (K^+^), and calcium (Ca^2^^+^) in various regions of the brain and spinal cord (7, 32). Among purinergic receptors, the P2X7 subtype has been shown to enhance glutamatergic excitation by promoting vesicular release from presynaptic terminals, as evidenced by its activation with 2′- and 3′-O-(4-benzoylbenzoyl)adenosine 5′-triphosphate, and inhibition by antagonists such as BBG or oxidized ATP in spinal cord slices (33). In pathological conditions such as SCI, significant amounts of ATP and other nucleotides are released from damaged tissue, which, in conjunction with growth factors, promotes lesion remodelling and repair (34). A limitation of this study is that the ATP biosensor measures total extracellular ATP and cannot distinguish regulated ATP release from passive leakage by damaged cells. Nevertheless, our previous studies showed that kainate induces an early increase in extracellular glutamate (through calcium-dependent and calcium-independent mechanisms) while LDH release remains low (<20% of total), suggesting limited membrane disruption (35). Thus, although a contribution from injured cells cannot be completely excluded, extracellular ATP remains a relevant danger signal associated with purinergic mechanisms involved in neuronal damage and repair (34).

Although the blockade of the P2X7 receptor with BBG has been proposed as a promising neuroprotective strategy following SCI the available evidence is mixed (36, 37). In contrast to the substantial functional recovery reported by Peng et al. (37), Marcillo et al. (38) found that systemic BBG administration did not significantly improve either motor function or histopathological outcomes. Consistent with the latter study, BBG co-treatment in our experimental conditions showed only modest behavioral effects, and *post hoc* analysis revealed no statistically significant difference between the KA and KA + BBG groups. Variations in experimental design, including differences in rat strain, injury level and severity, treatment paradigms, and outcome assessment methods, may contribute to these divergent results (38). It is also possible that the marked ATP release observed during the acute phase contributes to metabolic and cellular disturbances whose consequences become apparent only at later stages.

Our present data show that BBG, a non-nucleotide purinergic antagonist, exerts neuroprotective effects, as evidenced by a reduction in the number of pycnotic cells and the preservation of neuronal numbers. Although no significant modulation of astrocytic or microglial phenotypes was detected during this early post-KA phase, consistent with our previous observations within the same temporal window, it remains possible that ATP signalling and associated glial dynamics undergo substantial changes at later stages (26, 28). Therefore, alterations emerging during the subacute (~2 weeks) or chronic (~8 weeks) phases cannot be excluded and warrant future investigation. In line with previous findings (38), locomotor impairment was modest, and not significantly, reversed by BBG co-treatment in our study. While BBG primarily acts as a P2X7 receptor antagonist, it can also inhibit voltage-gated sodium (Nav) channels at experimentally relevant concentrations, albeit with lower potency (39). Therefore, a contribution of off-target Nav channel blockade to the observed effects cannot be completely excluded. One limitation of our study is the potential influence of genetic background on functional and cellular outcomes. Our *in vivo* experiments were conducted exclusively in BALB/c mice, whereas the *in vitro* preparations were obtained from either C57BL/6J or BALB/c mice. Previous evidence indicates that commonly used inbred strains can differ markedly in their locomotor recovery after SCI. Such strain-dependent variability may influence behavioral outcomes and should therefore be taken into account when interpreting the results of our study (23). This limitation also highlights the need for further preclinical studies to clarify the mechanisms underlying the limited efficacy observed.

Various pharmacological strategies targeting purinergic receptors are currently being explored for SCI and other neurological disorders (40). However, the clinical translation of P2X7 antagonists remains challenging due to interspecies differences in receptor pharmacology and variable efficacy across experimental models (41, 42). In the present study, BBG reduced neuronal loss but produced only limited functional improvement, highlighting the complexity of translating early neuroprotection into meaningful behavioral recovery.

In addition to P2X7, the transcriptional upregulation of P2X4 observed after KA treatment suggests that ATP-dependent signalling after excitotoxic injury is not restricted to a single purinergic receptor subtype. Although the functional contribution of P2X4 was not directly explored in the present study, previous reports have implicated this receptor in SCI-associated neuroinflammatory responses (17, 43–45). Future studies should determine whether P2X4 contributes to the early ATP-dependent mechanisms identified here and whether simultaneous targeting of P2X4 and P2X7 pathways provides greater neuroprotection.

Collectively, our findings support a central role for ATP and glutamate in the early excitotoxic cascade triggered by SCI. Their dysregulated release may contribute to neuronal loss and secondary injury processes, including the activation of inflammatory pathways and other bioactive mediators associated with progressive tissue degeneration (46–48). In addition to reducing neuronal loss, BBG treatment restored nuclear CREB immunoreactivity that was significantly decreased following KA exposure, suggesting that ATP-dependent signalling may influence transcriptional programs involved in neuronal survival. These findings are consistent with our previous observations linking altered CREB nuclear localization to excitotoxic injury (27). Although the specific molecular pathways connecting purinergic signalling and CREB regulation were not directly examined in the present study, our results identify CREB as a potential downstream target of ATP-mediated excitotoxic mechanisms and warrant further investigation into its contribution to neuroprotection after spinal cord injury.

Finally, the development of preclinical and translational neuroprotective and neuro-regenerative strategies, supported by animal models and complementary behavioral assessments, remains essential for advancing therapies that target the cellular and molecular mechanisms underlying SCI complications (49). In this context, ATP-gated ion channels, particularly the P2X7 receptor, have emerged as promising therapeutic targets because of their involvement in neuroinflammatory and excitotoxic processes (50). Consistent with this notion, our findings demonstrate that ATP is released early after KA-induced spinal cord excitotoxicity and contributes to neuronal vulnerability through purinergic signalling pathways. Although BBG reduced neuronal loss and partially preserved CREB nuclear translocation, its limited effect on motor recovery suggests that ATP-dependent mechanisms represent an important, but not exclusive, component of secondary injury progression. Further studies are needed to define the molecular mechanisms linking purinergic signalling to CREB-dependent pathways, including the potential regulation of CREB phosphorylation at Ser133. Further investigation of purinergic signalling may help identify therapeutic strategies that more effectively couple neuronal protection with functional recovery after spinal cord injury.

## 5 Conclusion

In summary, elucidating the early molecular and cellular responses to acute SCI is fundamental for guiding the development of targeted neuroprotective strategies. Our findings provide preclinical evidence that early ATP release following excitotoxic damage is associated with changes in neuronal survival and network plasticity. The observed alterations in CREB-dependent signalling pathways may represent a cellular response linked to ATP-mediated excitotoxic injury. However, a limitation of this study is that the association between CREB-dependent signalling and neuronal loss does not establish a causal role for CREB signalling in injury progression. Although both *in vitro* and *in vivo* results are encouraging, further studies are required to evaluate whether modulation of ATP-mediated signalling can provide sustained neuroprotection after SCI. Continued investigation of ATP-driven mechanisms may facilitate the development of targeted therapeutic strategies for SCI-related complications.

## Data Availability

The raw data supporting the conclusions of this article will be made available by the authors, without undue reservation.

## Footnotes

1
http://www.ncbi.nlm.nih.gov/tools/primer-blast/

## Appendix

### Resource identification initiative

**Table A1:** Key resources table (RRIDs).

Resource	Source	Catalog number	RRID
Mouse strain: C57BL/6J	—	—	RRID: IMSR_JAX:000664
Mouse strain: BALB/c	—	—	RRID: IMSR_JAX:000651
Anti-CREB	Cell Signaling Technology	9,197 (48H2)	RRID: AB_331277
Anti-NeuN	Merck Millipore	MAB 377	RRID: AB_2298772
Anti-S100β	Dako (Agilent)	Z0311	RRID: AB_10013383
Anti-Iba1	Wako	019–19741	RRID: AB_839504
Anti-Iba1	Abcam	ab178847	RRID: AB_2832244
Goat anti-mouse IgG	Jackson ImmunoResearch	115–095-003	RRID: AB_2338589
Donkey anti-rabbit IgG	Jackson ImmunoResearch	711–025-152	RRID: AB_2340588
ImageJ	NIH	—	RRID: SCR_003070
GraphPad Prism	GraphPad Software	—	RRID: SCR_002798
